# Synthesis of lignin based composites of TiO_2_ for potential application as radical scavengers in sunscreen formulation

**DOI:** 10.1186/s13065-019-0537-3

**Published:** 2019-02-04

**Authors:** Mohamad Nasir Mohamad Ibrahim, Anwar Iqbal, Chai Chuan Shen, Showkat Ahmad Bhawani, Farook Adam

**Affiliations:** 1 School of Chemical Sciences, Universiti Sains Malaysia, 11800 Gelugor, Penang Malaysia; 2 Department of Chemistry, Faculty of Resource Science and Technology, Universiti Malaysia Sarawak (UNIMAS), 94300 Kota Samarahan, Sarawak Malaysia

**Keywords:** TiO_2_, Hydroxyl radical, Lignin, Oil palm empty fruit bunch, Lignin/TiO_2_ composite

## Abstract

Titanium dioxide (TiO_2_) is added in sunscreens due to its ability to absorb ultraviolet (UV) light. However, upon irradiation of UV light, reactive oxygen species particularly hydroxyl radical which can damage human skin will be generated. In this study, lignin/TiO_2_ composites were employed to quench the hydroxyl radicals generated by the TiO_2_. The lignin was extracted from oil palm empty fruit bunch (OPEFB) via kraft and soda pulping processes. The kraft lignin composite was labelled as KL/TiO_2_ whereas the soda lignin composite was labelled as SL/TiO_2_. The lignins and the composites were characterized by FTIR, UV spectroscopy, ^13^C NMR, SEM, EDX, and XRD. The relative hydroxyl radical production of composites and TiO_2_ were compared through photo-oxidation of coumarin to 7-hydroxycoumarin as a test medium. The effect of types and amounts of lignin used were studied. The KL/TiO_2_ composite showed the least radical production due to higher phenolic hydroxyl content of kraft lignin. The activity of the hydroxyl radicals will be quenched when it abstract hydrogen atoms from the phenolic hydroxyl groups.

## Introduction

Titanium dioxide (TiO_2_) is used as an inorganic agent in sunscreens due to its ability to reflect, scatter and absorb a wide range of ultraviolet radiation in sunlight [1]. The maximum loading of TiO_2_ in sunscreens is 25%, and the crystalline form of TiO_2_ that mostly used for this application is anatase [2]. The TiO_2_ is also employed as opacifiers and pigments in paints and paper coatings due to the whiteness and opaque characteristics [3]. Moreover, TiO_2_ possesses excellent photocatalytic capability which makes it suitable for removal of organic compounds in contaminated water [4]. However, this capability of TiO_2_ is a double-edged sword. The photocatalytic activity of TiO_2_ can generate superoxide and hydroxyl radicals by the irradiation of sunlight. This is unfavorable for the application in sunscreens. These reactive oxygen species with cytotoxic and genotoxic characteristics can react with biomolecules such as protein and DNA upon formation on the surface of TiO_2_ [5]. This may result in carcinogenesis, enzyme inactivation and potential damage of biomolecules [5]. The reactive oxygen species will cause the degradation of other organic agents in sunscreen [6].

A variety of methods have adopted by many researchers to reduce the photocatalytic activity of TiO_2_. The alumina was used to coat TiO_2_ by Picatonotto et al. [7]. The inorganic surface coating minimizes the photocatalytic activity by reducing holes and electrons generation and enhancing electron–hole recombination which leads to quenching of photocatalytic activity. The encapsulation of TiO_2_ in zeolites reduced its photocatalytic efficiency by increasing the band gap so that excitation can only take place under ultraviolet radiation with the wavelength below 265 nm (ultraviolet C (UVC) region) [8]. The UVC can be absorbed by the ozone layer and will not reach the surface of the earth. Moreover, instead of inhibiting the photocatalytic activity of TiO_2_, Chen et al. [9] utilized the antioxidant properties of chitosan to capture the free radicals generated by the photocatalytic activity of TiO_2_.

**Fig. 1:**
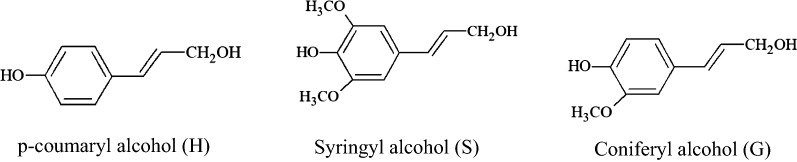
The structure of three major phenylpropanoid units of lignin

Chemically, lignin is a natural, amorphous and cross-linked phenolic biopolymer with very complex structure [10]. Lignin provides structural rigidity for plants and supports transport of nutrients and water in plants. It can be extracted from the black liquor which is always considered as waste in pulping and paper industries [11]. Lignin is made up of three major phenylpropanoid units that differ in the amount of methoxyl groups namely *p*-hydroxyphenyl (H), guaiacyl (G) and syringyl (S) as shown in Fig. 1 [10]. It can be extracted from different biomass materials such as oil palm ligocellulosic waste, and kenaf through soda, kraft, ethanol or other pulping processes [10, 12]. The composition and properties of lignin vary for different pulping processes, types, and parts of plants.

The oil palm empty fruit bunch (OPEFB) is one of the agriculture wastes generated by the oil palm industry. The oil palm agriculture wastes are normally burned in incinerators and thus lead to environmental pollutions such as haze which affect human health [13]. In the previous studies, OPEFB lignin has been employed in several applications such as food emulsifying agent, heavy metal adsorption for waste water treatment, wood adhesive, corrosion inhibitor and additives in mud drilling [14]. The special advantage of OPEFB lignin is that it can be obtained from agriculture wastes instead of other plants such as pine tree and acacia tree which may involve deforestation.

The previous study showed that oil palm empty fruit bunch lignin exhibited antioxidant properties and acted as free radical scavenger due to the presence of phenolic hydroxyl group [15]. The phenolic hydroxyl groups act as a proton donor and can stabilize the resulting radicals by substituents and delocalization. Besides the antioxidant properties, the cytotoxic effects of lignin from different sources have been studied and lignin from all sources are proved safe to the human cell [16]. This previous research has removed the doubts about the safety concerns of lignin. Lignin can be a natural sun blocker for broad spectrum since it contains a large amount of ultraviolet absorbing functional groups such as phenolic, ketone and other chromophores as reported by Qian et al. [17].

The antioxidant properties and UV absorption capability of lignin had motivated us to employ lignin to scavenge the hydroxyl radicals generated from the photocatalytic activity of TiO_2_ by forming a lignin/TiO_2_ composite. The effects of types and amounts of lignin were investigated. Although lignin has been used for reducing phototoxicity of TiO_2_ by some researches [2, 18], the lignin extracted from oil palm empty fruit bunch for scavenging free radical generated by TiO_2_ has not been explored yet. Therefore, this study also intends to recycle the oil palm lignocellulosic waste into a useful product for potential cosmetic application. Thus, a disposal alternative of agriculture wastes in the oil palm industry is suggested.

## Experimental

### Materials

The oil palm empty fruit bunch (OPEFB) fiber used in this study was supplied by Sabutek (M) Sdn. Bhd., Malaysia, a local company specializing in recycling oil palm lignocellulosic waste. All the chemicals used are analytical reagent grade. Most of them are from Qrec (Asia) Sdn. Bhd., Malaysia and Sigma-Aldrich Chemie GmbH, Germany.

### Kraft and soda pulping processes

Both kraft and soda pulping processes were carried out in a 10 L stainless steel rotary digester. The OPEFB (500 g) fiber was used for both pulping processes. For kraft pulping, 19% of active alkali and 25% of sulfidity with water to fiber ratio of 8 were added to the digester. The digester was heated from room temperature to 170 °C for 1 h and maintained at 170 °C for another 3 h. For soda pulping, 25% of active alkali with no percentage of sulfidity was added into the digester and similar conditions as kraft pulping were used [15]. The black liquor produced from the pulping processes was separated from the pulp by filtering with screening through a filtering sieve twice and collected. The black liquor was filtered again via vacuum filtration for further removing the pulp from black liquor.

### Preparation of OPEFB Kraft and Soda Lignin

The pH of kraft and soda black liquors measured were 13.75 and 13.49, respectively. The kraft and soda lignins were precipitated from concentrated black liquors by acidifying them until pH 2.0 using 20% (v/v) sulfuric acid. The suspensions were centrifuged at 3500 rpm for 10 min. The liquid layer was discarded while the precipitate was washed with acidified distilled water (pH 2.0). The kraft and soda lignins were dried in an oven at 55 °C for 3 days [15].

### Synthesis of lignin/TiO_2_ composites

The lignin/TiO_2_ composites were synthesized by using the previously reported method [18]. The kraft lignin (1.0 g) was mixed with 70 mL of ultrapure water under stirring. The solubilization of kraft lignin was carried out by adding 30 mL of 1 M NaOH dropwise followed by the addition of 0.1 g of anatase TiO_2_. The mixture was homogenized at 900 rpm for 40 min and sonicated at 40 kHz for 150 min. The mixture was cooled down to 0 °C in an ice bath and diluted with 200 mL of ultrapure water. The kraft lignin/TiO_2_ composite was precipitate by adding 300 mL of 0.25 M. The kraft lignin/TiO_2_ suspension was centrifuged at 3500 rpm for 10 min. The liquid layer was discarded. The precipitate was washed with ultrapure water twice and dried in an oven at 80 °C for 6 h. The dried kraft lignin/TiO_2_ composite was ground into powder and labeled as KL/TiO_2_-1.0. The procedures were repeated by using soda lignin to produce soda lignin/TiO_2_ composite. The composite was labelled as SL/TiO_2_-1.0. The other three composites were prepared by varying the weight of kraft lignin added (0.5 g, 1.5 g, and 2.0 g). The kraft lignin/TiO_2_ composites formed were labeled as KL/TiO_2_-0.5, KL/TiO_2_-1.5 and KL/TiO_2_-2.0, respectively.

### Characterization of Lignin and Lignin/TiO_2_ Composite

#### FTIR analysis

The FTIR analysis was performed for lignins, lignin/TiO_2_ composites and anatase TiO_2_ by using Perkin Elmer model System 2000 instrument. The FTIR spectra were recorded in a direct transmittance mode and a frequency range of 4000 to 400 cm^−1^ with a resolution of 4 cm^−1^ and 16 scans. The KBr pellet for FTIR analysis was prepared by mixing the solid sample with KBr in a ratio of 1:100 (w/w).

#### UV spectroscopy

The UV spectrometric analysis of kraft and soda lignins in dioxane solution and alkaline solution were conducted. In brief, 7 mg of lignin was dissolved in 5 mL of dioxane-water solution (9:1, v/v). Similarly, 7 mg of lignin was dissolved in 5 mL of pH 12 NaOH solution. Subsequently, 50-fold dilution was carried out for both solutions [10]. The UV spectra were recorded by using UV Perkin Elmer Lambda 35.

#### ^13^C NMR analysis

Since lignin is difficult to dissolve in most deuterated solvents [12], acetylation was done to enhance the results of the analysis. 1.0 g of lignin, 7.5 mL pyridine and 7.5 mL acetic anhydride were mixed in 250 mL conical flask and stirred for 24 h at room temperature. The mixture was cooled down to 0 °C in an ice bath. Into the solution, 150 mL of 1% HCl (w/v) was then added dropwise. The formed precipitate was filtered using vacuum filtration. The resulting precipitate was washed with distilled water and dried in an oven at 50 °C for 48 h. The acetylated lignin (150 mg) was dissolved in 0.4 mL d_6_-DMSO and injected into the NMR tube. The analysis was conducted at 50 °C with 22,000 scans by using Bruker Avance 500 MHz.

#### XRD analysis

The XRD analysis was performed for KL/TiO_2_-1.0 and SL/TiO_2_-1.0 using Bruker D8 Advance equipped with Cu Kα radiation, λ of 0.154 nm, the voltage of 40 kV and current of 40 mA. The XRD diffractogram were recorded with 2 theta degree ranged from 10°to 90° at a continuous scanning rate of 3° min^−1^.

#### SEM and EDX analysis

The SEM analysis was carried out for kraft lignin, KL/TiO_2_-1.0 and SL/TiO_2_-1.0 and anatase TiO_2_ with Leo Supra 50 VP Field Emission Scanning Electron Microscope. The magnification was set as 10,000 for each sample. The EDX analysis was conducted for KL/TiO_2_-1.0 and SL/TiO_2_-1.0 using Oxford-Instruments INCA 400 with X-Max Detector.

### Detection of hydroxyl radicals

Coumarin solution was used to detect the presence of hydroxyl radicals following the method reported by Ishibashi et al. [19] using Perkin Elmer LS 55 Fluorescence Spectrophotometer with the excitation wavelength of 332 nm. Coumarin powder (0.15 g) was dissolved in 1 L distilled water and sonicated at 40 kHz for 2 h to prepare 1 × 10^−3^ M coumarin solution. A total of five coumarin solutions were prepared by adding kraft lignin, soda lignin, KL/TiO_2_-1.0, SL/TiO_2_-1.0, and anatase TiO_2_, respectively. All the coumarin solutions were shaken at 500 rpm for 15 min before irradiated under sunlight. The sample was withdrawn from the coumarin solution and filtered with a 0.45 µm syringe filter every 15 min interval. The sample (0.5 mL) was diluted to 10 mL with distilled water. The diluted sample was used to determine the fluorescence intensity which indirectly corresponds to the amount of hydroxyl radicals produced. The procedures were repeated with KL/TiO_2_-0.5, KL/TiO_2_-1.0, KL/TiO_2_-1.5, and KL/TiO_2_-2.0.

## Results and discussion

### FTIR analysis

**Fig. 2:**
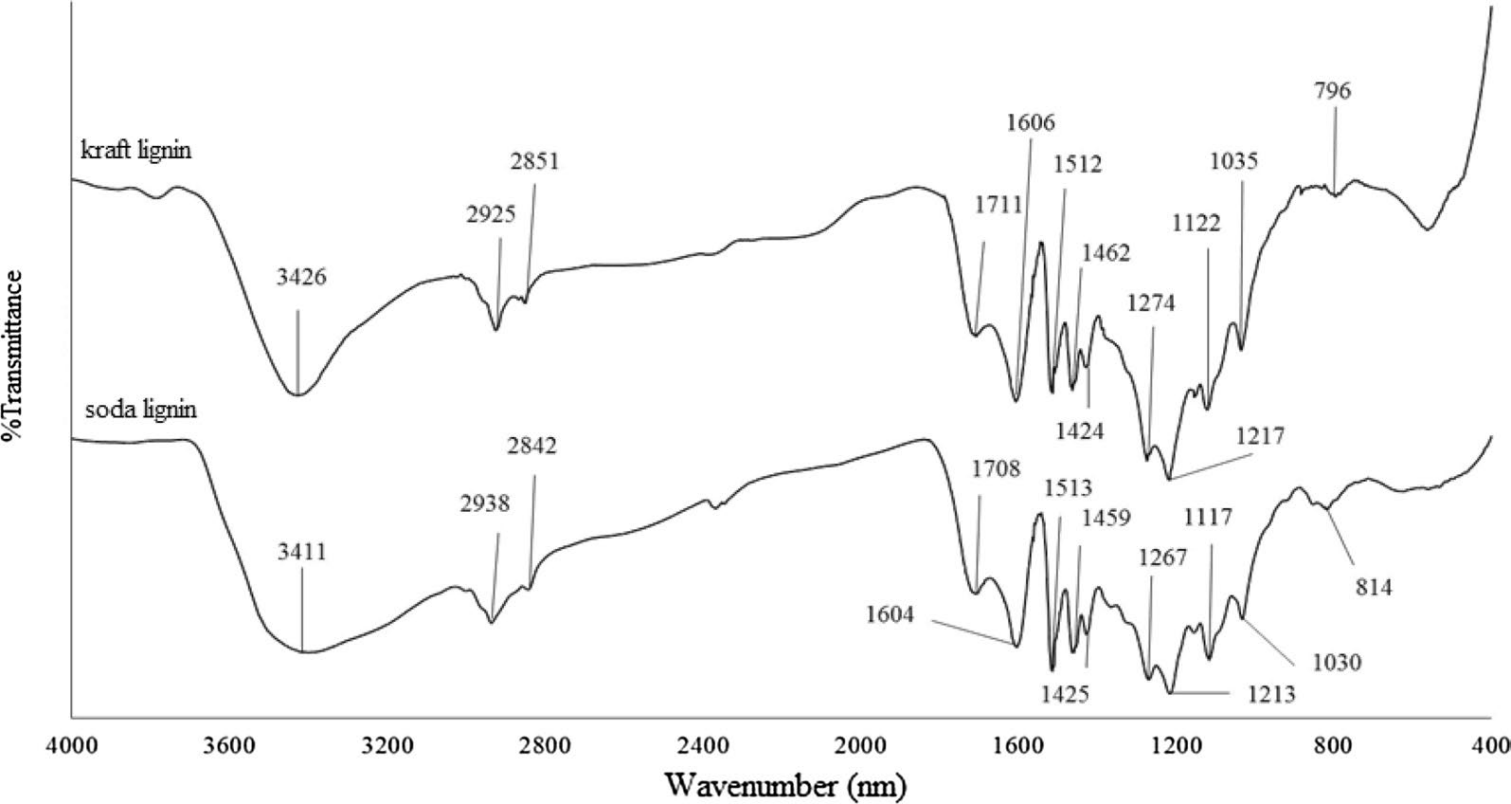
FTIR spectra of kraft lignin and soda lignin with wavenumber of 4000 cm^−1^ to 400 cm^−1^

Figure 2 shows the FTIR spectra for kraft and soda lignins. The corresponding assignments and bands for both lignins are presented in Table 1. The assignments of the bands are reported according to previous literature finding [10]. It was revealed that kraft and soda lignins had similar functional groups. This finding is in agreement with the results reported by Ibrahim et al. [10]. A wide absorption band which appeared around 3400 cm^−1^ is assigned to aromatic and aliphatic OH groups. The absorption bands in the region between 3000 and 2840 cm^−1^ are attributed to C-H stretching in methyl, methylene and methoxyl groups. The absorption band between 1715 and 1705 cm^−1^ presented in the spectra of both lignins can be related to the unconjugated carbonyl stretching. Both kraft and soda lignins showed absorption bands around 1600 cm^−1^, 1515 cm^−1,^ and 1425 cm^−1^ which are attributed to typical aromatic ring vibrations of the phenylpropane (C9) skeleton. The absorption band around 1460 cm^−1^ attributes to C–H deformation in methyl, methylene and methoxyl groups. The absorption band around 1270 cm^−1^ and 1117 cm^−1^ in the spectra can be attributed to C–O stretching vibration of a secondary alcohol and aromatic C–H in plane deformations of syringyl, respectively. The absorption band presented around 1220 cm^−1^ is assigned to C–O stretching in syringyl (S) and guaiacyl (G) which also indicates the presence of phenolic hydroxyl group and ether in syringyl and guaiacyl. The absorption band around 1030 cm^−1^ corresponds to the aromatic C–H in-plane deformation of guaiacyl. The aromatic C–H out of plane bending appears at 796 cm^−1^ and 814 cm^−1^ in the spectra for kraft lignin and soda lignin respectively.

**Table 1:** FTIR absorption bands and assignments for kraft and soda lignins

Frequency (cm^−1^) [10]	Assignments	Frequency (cm^−1^)	
		Kraft	Soda
3450–3400	O–H stretching (phenolic OH and aliphatic OH)`	3426	3411
2960–2925	C–H stretching (CH3 and CH_2_ groups)	2925	2938
2855–2840	C–H stretching (OCH_3_)	2851	2842
1715–1705	C=O stretching (unconjugated ketone, carbonyl and ester groups)	1711	1708
~ 1600	C–C stretching (aromatic skeleton)	1606	1604
1513	C–C stretching (aromatic skeleton)	1512	1513
1460	C–H deformation (asymmetric in –CH_3_ and –CH_2_)	1462	1459
1425	C–C stretching (aromatic skeleton) with C-H in-plane deformation	1424	1425
1270	C–O stretching vibration of secondary alcohol	1274	1267
~ 1220	C–O(H) + C–O (Ar) (phenolic OH and ether in syringyl and guaiacyl)	1217	1213
1115	Ar–CH in plane deformation (syringyl)	1122	1117
~ 1030	C–O(H) + C–O(C) (first order aliphatic Oh and ether	1035	1030
815–795	C–H out of plane (aromatic ring)	796	814

**Fig. 3:**
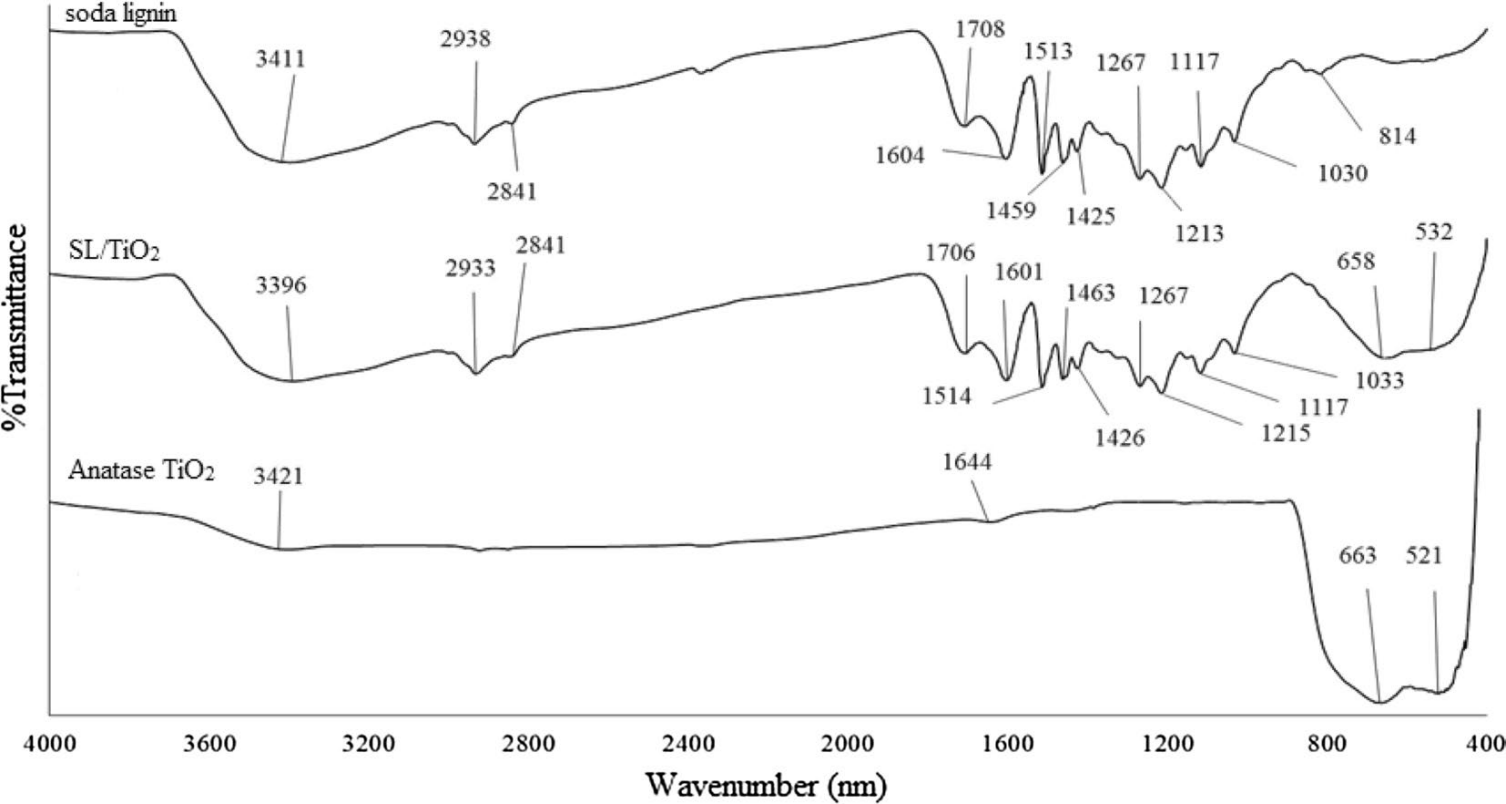
FTIR spectra of soda lignin, SL/TiO2-1.0 and anatase TiO2 with wavenumber of 4000 cm^−1^ to 400 cm^−1^

**Fig. 4:**
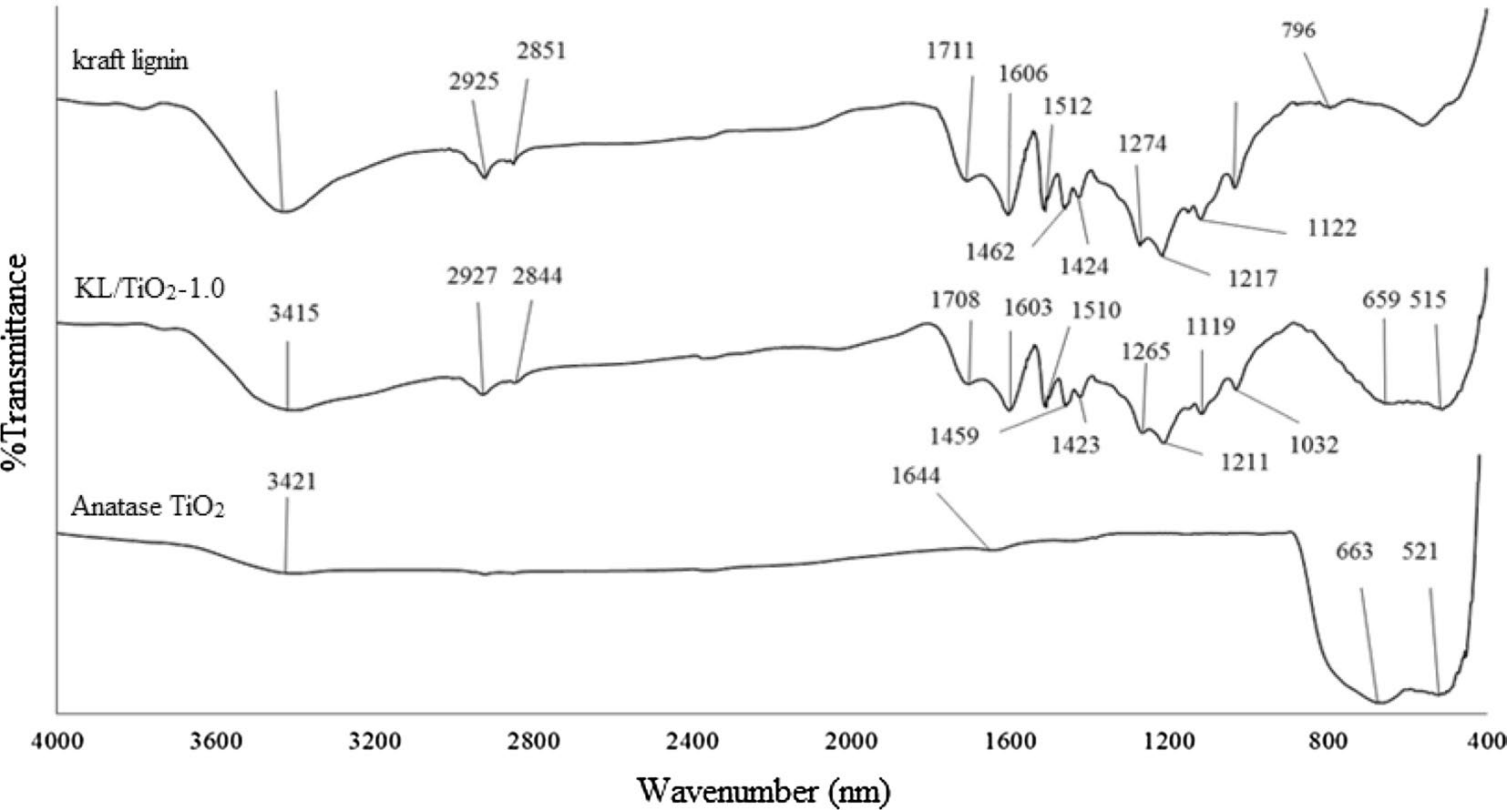
FTIR spectra of kraft lignin, KL/TiO2-1.0 and anatase TiO2 with wavenumber of 4000 cm^−1^ to 400 cm^−1^

The absence of absorption band at 1166 cm^−1^ in the spectra which is normally assigned to *p*-hydroxyl phenylpropane indicates that OPEFB lignin is more similar to wood lignin instead of plant lignin which is normally HGS lignin. The FTIR spectra of lignin/TiO_2_ composites are shown in Figs. 3 and 4. Both composites, KL/TiO_2_-1.0, and SL/TiO_2_-1.0 have far more absorption bands between 4000 and 700 cm^−1^ compared to anatase TiO_2_ due to different functional groups and complex cross-linked phenolic structure in lignin. Besides, both composites showed typical sharp peaks around 663 cm^−1^ and 521 cm^−1^. These peaks are attributed to Ti–O-Ti stretching bonds [2]. The composites exhibited the absorption bands corresponding to both lignin and TiO_2_ indicating that lignin has successfully formed composite with TiO_2_.

**Scheme 1:**
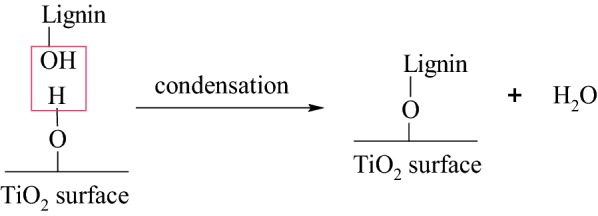
The condensation reaction betwen hydroxyl groups of lignin and surface hydroxyl groups of TiO_2_

The surface of TiO_2_ and lignin molecules have abundant hydroxyl groups. Through condensation reaction, these hydroxyl groups can react with each other and form linkages as shown in Scheme 1. The bond formation is possible since the synthesis is carried in one pot route. Water is removed as the second product.

### UV spectroscopy

**Fig. 5:**
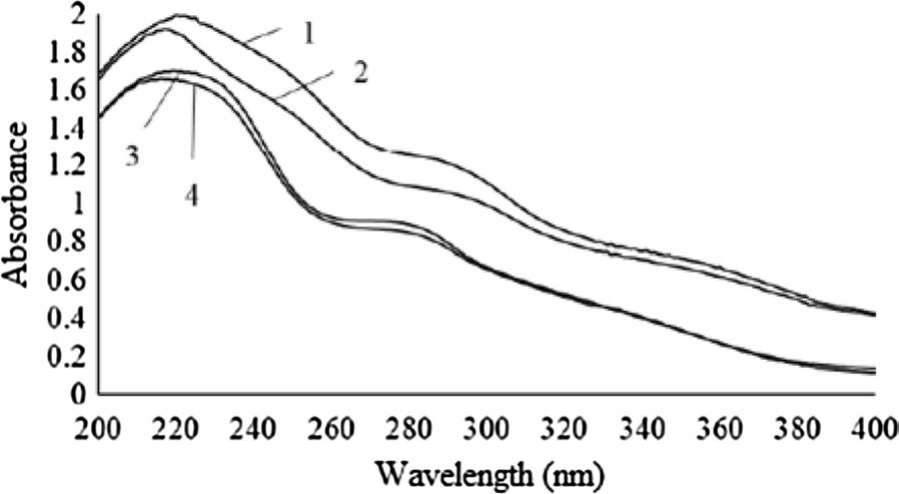
UV spectra of kraft lignin and soda lignin. 1—kraft lignin in pH 12 NaOH solution; 2—soda lignin in pH 12 NaOH solution; 3—kraft lignin in dioxane-water (9:1, v/v) solution; 4—soda lignin in dioxane-water (9:1, v/v) solution

The non-etherified phenolic group is the most important functional group in lignin. It is because the phenylpropane units are most likely to be attacked in the chemical reactions of lignin. In this study, the phenolic groups presented in both kraft and soda lignins was determined by UV spectroscopy and ^13^C NMR [10, 12]. Figure 5 shows the UV spectra of kraft lignin and soda lignin in neutral solution and alkaline solution which was a dioxane-water solution (9:1, v/v) and pH 12 NaOH solution respectively. The absorption around 280 nm can be attributed to non-conjugated phenolic units [19]. Both lignins showed first maximum absorption around 220 nm and second maximum absorption around 280 nm in dioxane-water solution. However, in pH 12 NaOH solution, the two lignins showed two maximum absorption around 220 and 290 nm. The maximum absorption of kraft lignin and soda lignin were shifted to 291 nm and 292 nm respectively in pH 12 NaOH solution as shown in Table 2.

**Table 2:** λ_max_ around 280 nm for soda lignin and kraft lignin

Lignin	λ_max_ in dioxane-water solution (nm)	λ_max_ in pH 12 NaOH solution (nm)
Kraft	282	291
Soda	283	292

This is mainly due to the dissociation of phenolic group in alkaline solution increases the conjugation of the oxygen atom with the benzene ring and thus shifts the maximum absorption to a higher wavelength. The absorbance of kraft lignin was higher than that of soda lignin in both dioxane-water solution and pH 12 NaOH solution. This can be explained by the fact that kraft lignin contains higher phenolic hydroxyl content than soda lignin that increases the electron density in benzene ring and absorbance around 280 nm.

### ^13^C NMR spectroscopy

**Fig. 6:**
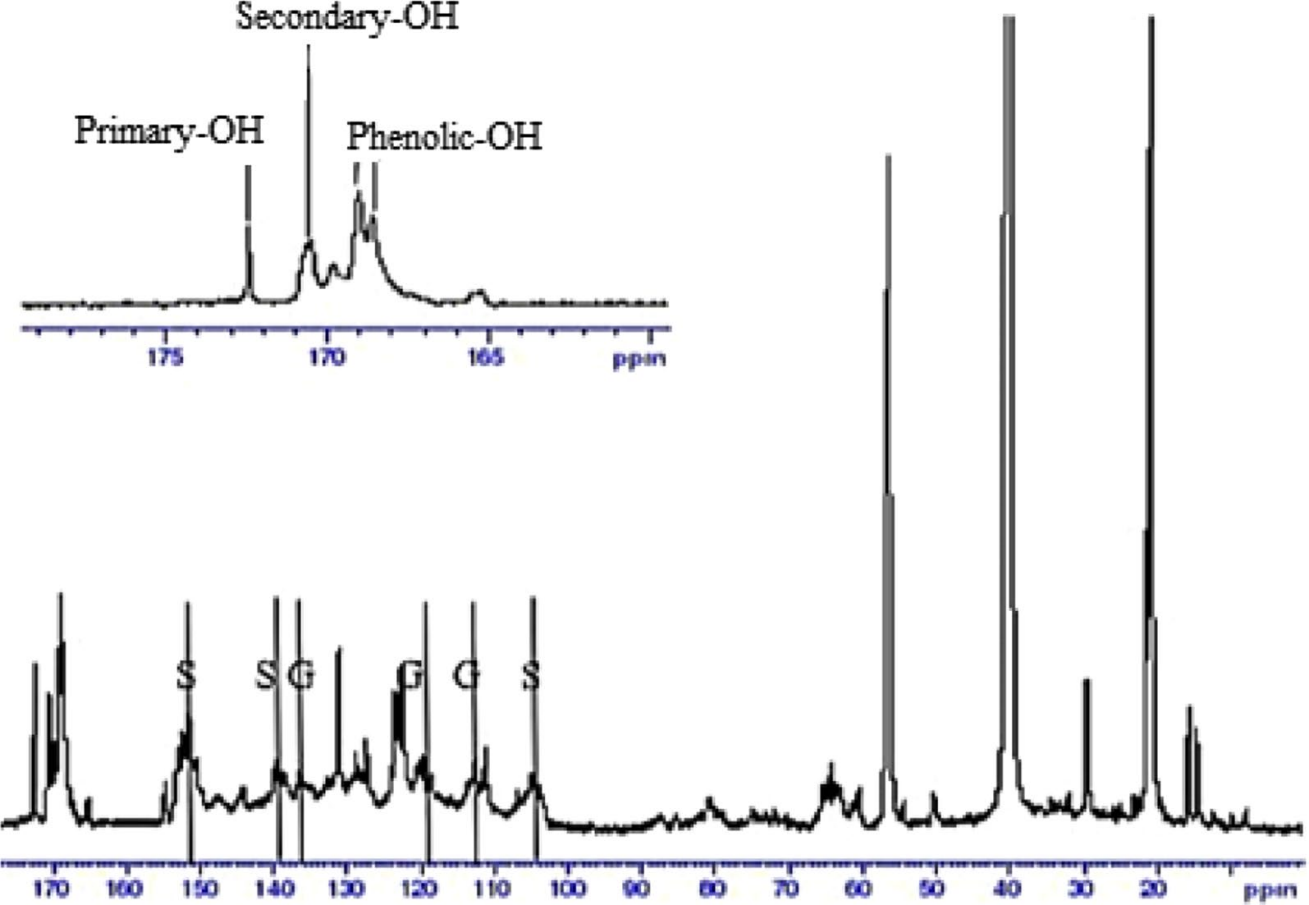
^13^C NMR spectrum of kraft lignin

**Fig. 7:**
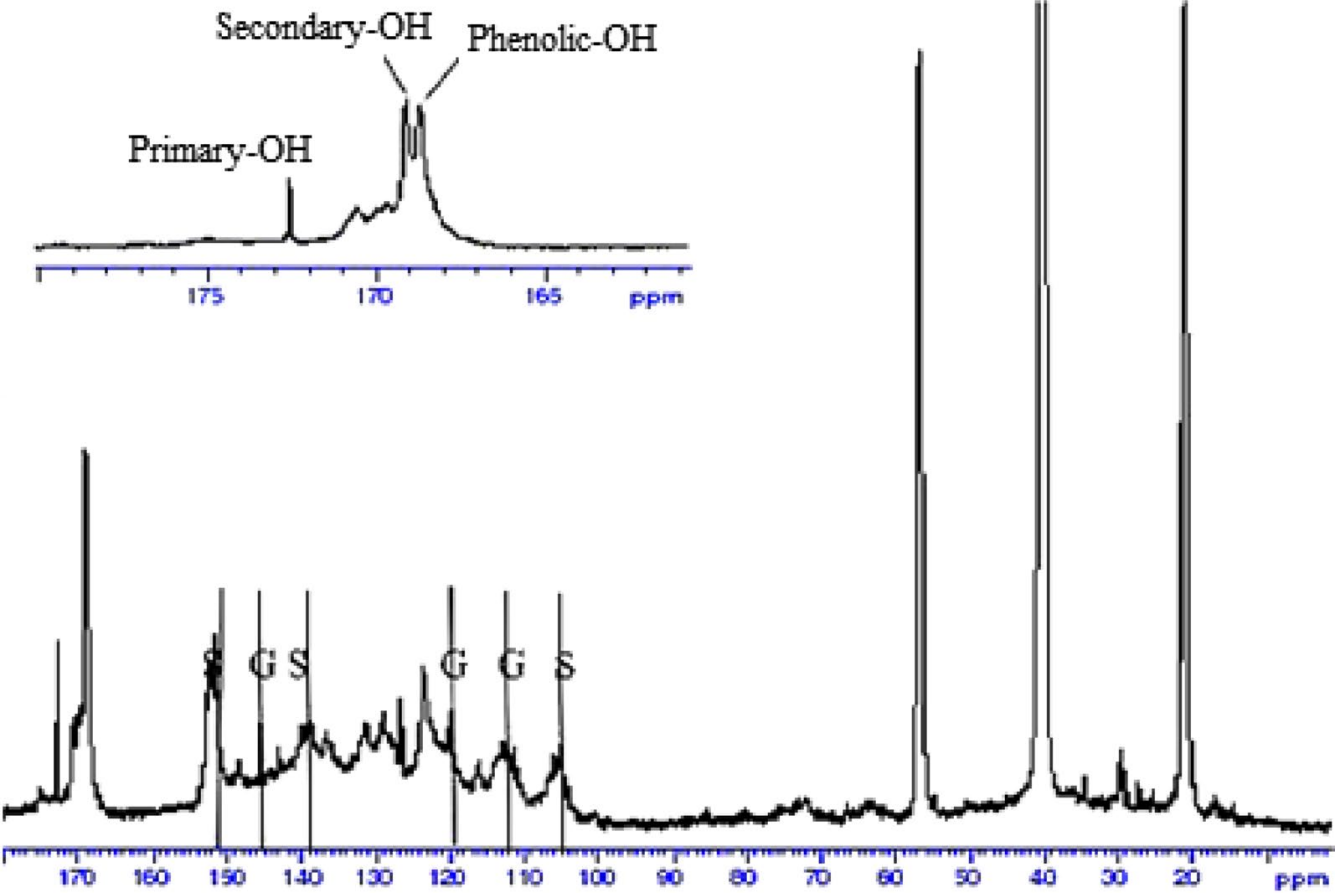
^13^C NMR spectrum of soda lignin

The results of UV analysis was supported by ^13^C NMR analysis. The estimation of lignin moieties was carried out by referring to Capanema et al. [20]. The ^13^C NMR spectra of acetylated kraft and soda lignins are shown in Figs. 6 and 7 respectively. The integral of the 155–102 ppm region was set as a reference and assumed that it includes six aromatic carbons and 0.12 vinylic carbons. The integral values were divided by 6.12 which is equivalent to one aromatic ring (Ar). It was observed that kraft lignin contained higher amount of secondary aliphatic –OH (0.35 Ar^−1^) than that of soda lignin (0.26 Ar^−1^) which was determined from the integration from 170 to 169 ppm region respectively. It was revealed that the amount of primary aliphatic –OH for kraft lignin (0.30 Ar^−1^) was higher than soda lignin (0.28 Ar^−1^) according to the integration from 173 to 170 ppm. On the other hand, kraft lignin consisted of a larger quantity of phenolic –OH (0.67 Ar^−1^) than soda lignin (0.46 Ar^−1^) based on the integration from 169 to 167 ppm. This can be contributed to the more intense cleavage of alkyl–aryl ether linkages during the kraft pulping process which lead to the formation of more phenolic end groups and thus increased the phenolic hydroxyl content in kraft lignin [16]. The higher phenolic –OH content enabled more hydroxyl radicals can be neutralized by the hydrogen atom abstraction from phenolic hydroxyl group.

Both soda and kraft lignins showed signals at 104 ppm, 138.5 ppm and 152 ppm which can be attributed to C-2 or C6, C-4, and C-3 or C5 in S unit. Besides, both lignins also gave signals at 112 ppm and 119 ppm which can be assigned to C-2 and C-6 in G unit. The higher S unit signals in both lignins are owning to OPEFB lignin contained more S unit compared to G unit. The signals at 123.5 ppm and 131 ppm can be referred to pyridine which was used for acetylation of lignin [21].

### XRD analysis

**Fig. 8:**
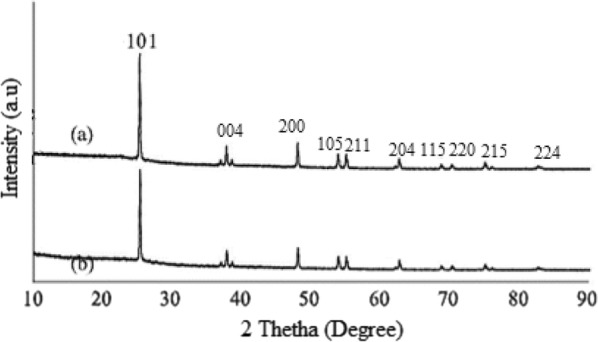
XRD diffractograms of a SL/TiO2-1.0 and b KL/TiO2-1.0 composites

The crystal structure of KL/TiO_2_-1.0 and SL/TiO_2_-1.0 were investigated through XRD analysis. The XRD diffractograms in Fig. 8 reveal that both composites had similar XRD patterns to that of pure anatase TiO_2_ [22]. The diffraction peaks at 2θ = 25.4°, 37.9°, 48.1°, 53.9°, 55.1°, 62.7°, 68.8°, 70.4°, 75.1° and 82.7° can be indexed to (101), (004), (200), (105), (211), (204), (115), (220), (215) and (224) crystal planes of anatase TiO_2_, respectively. This indicates that the initial crystal cell structure of TiO_2_ is maintained and no other crystalline by-products are formed even after the formation of lignin/TiO_2_ composite. Since lignin is amorphous in nature and lack of ordered structure [10, 12], the XRD diffractograms of both composites did not show any diffraction peak that can be referred to lignin.

### SEM and EDX analysis

**Fig. 9:**
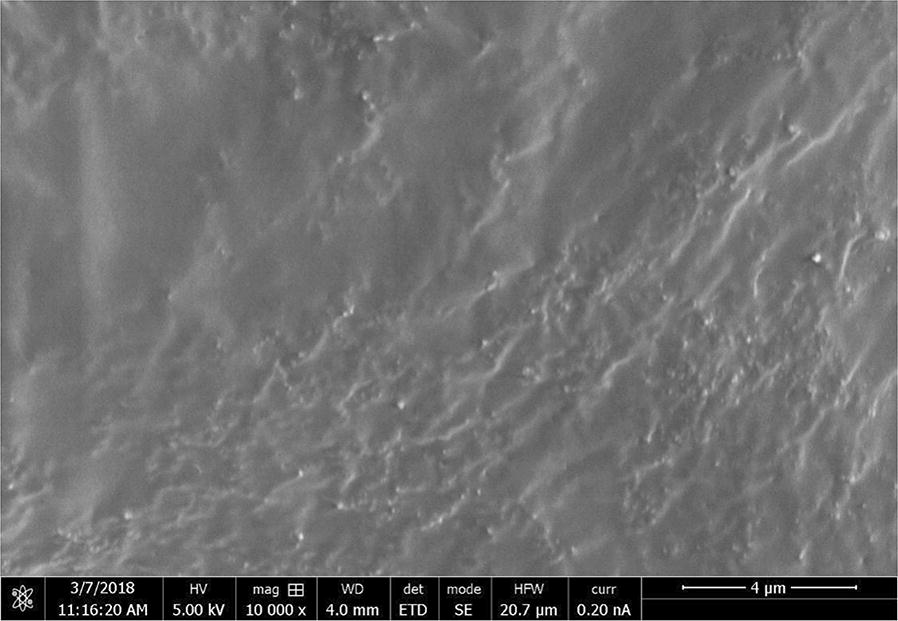
SEM image of anatase TiO_2_ at 10,000×  magnification

**Fig. 10:**
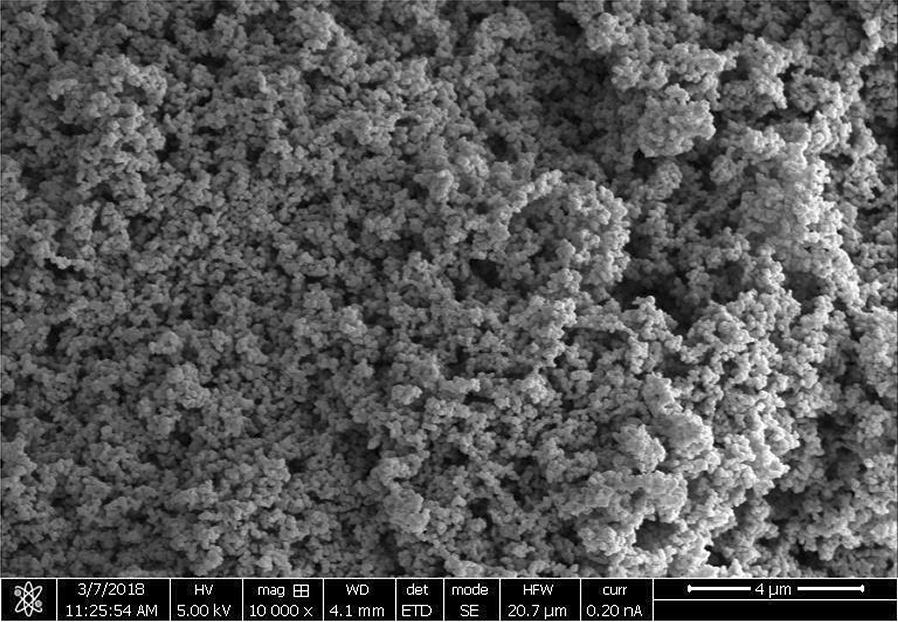
SEM image of kraft lignin at 10,000× magnification

**Fig. 11:**
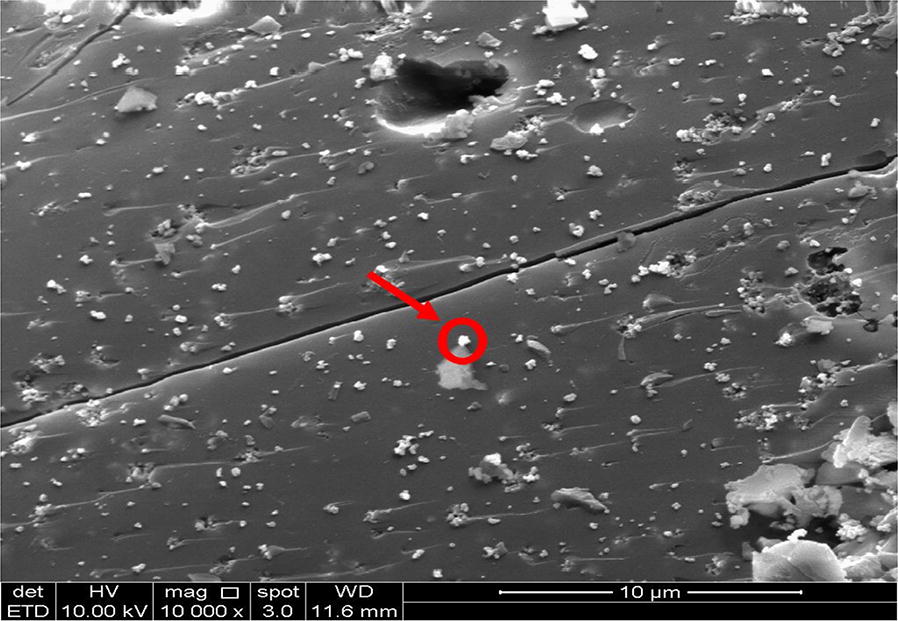
SEM image of KL/TiO_2_-1.0 at 10,000×  magnification

**Fig. 12:**
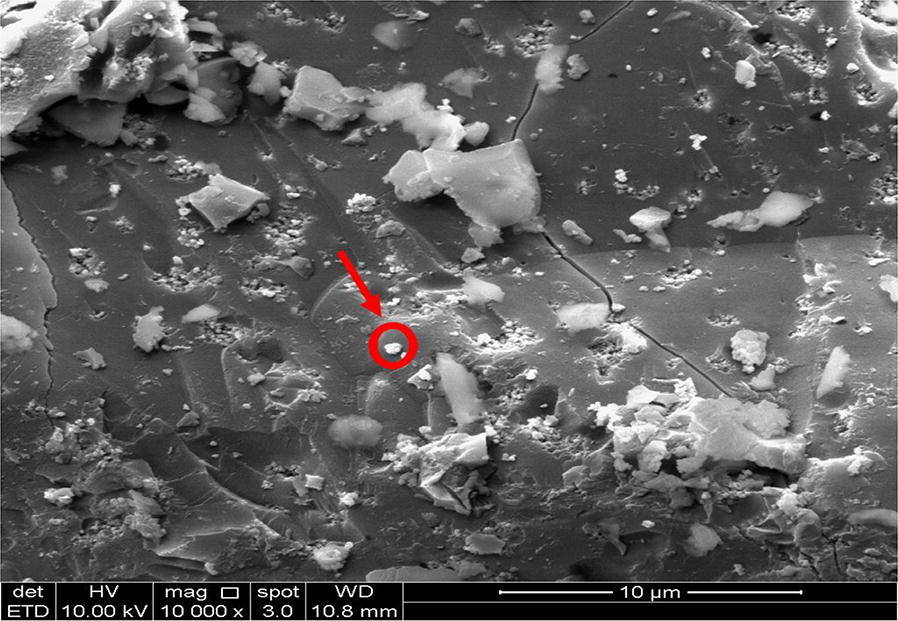
SEM image of SL/TiO_2_-1.0 at 10,000×  magnification

The SEM analysis was carried out to study the surface morphologies of kraft lignin, KL/TiO_2_-1.0, SL/TiO_2_-1.0, and anatase TiO_2_. The micromorphology of TiO_2_ was presented as many small spherical particles tended to aggregate to form a large sphere as shown in Fig. 9. Figure 10 shows that kraft lignin possessed typical rough surface morphology of lignin [11]. Figures 11 and 12 depict the surface of KL/TiO_2_-1.0, and SL/TiO_2_-1.0 were rougher than and significantly different from the surface morphology of pure lignin and crystal morphology of naked TiO_2_. Based on Figs. 11 and 12, it was found that TiO_2_ particles were well dispersed in the lignin matrix indicating TiO_2_ is incorporated into lignin successfully. Besides, the tendency of TiO_2_ particles to aggregate was reduced in both composites compared to that in pure anatase TiO_2_. This is mainly because of the sonication used in the formation of composite prevents the aggregation of TiO_2_ particles.

**Fig. 13:**
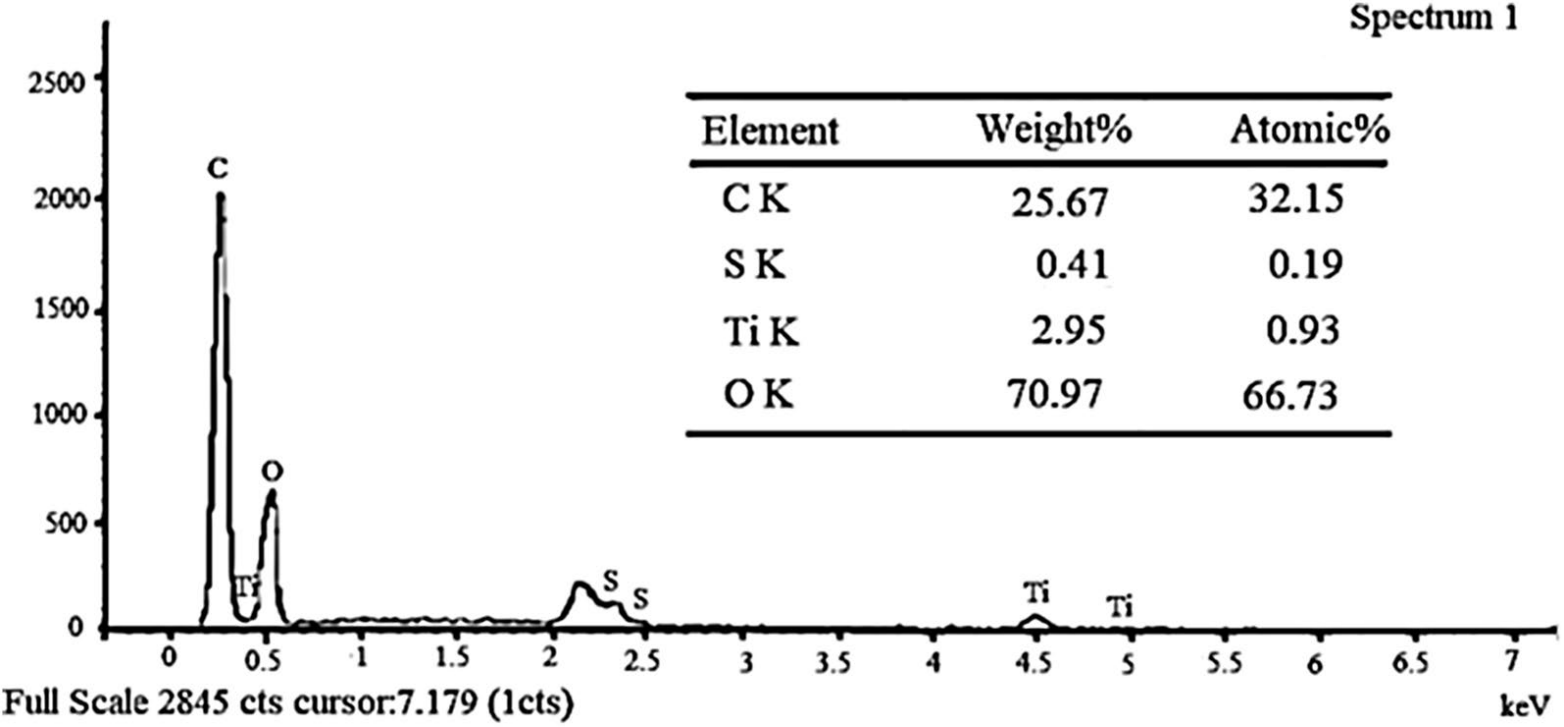
EDX analysis of KL/ TiO_2_-1.0 composite

**Fig. 14:**
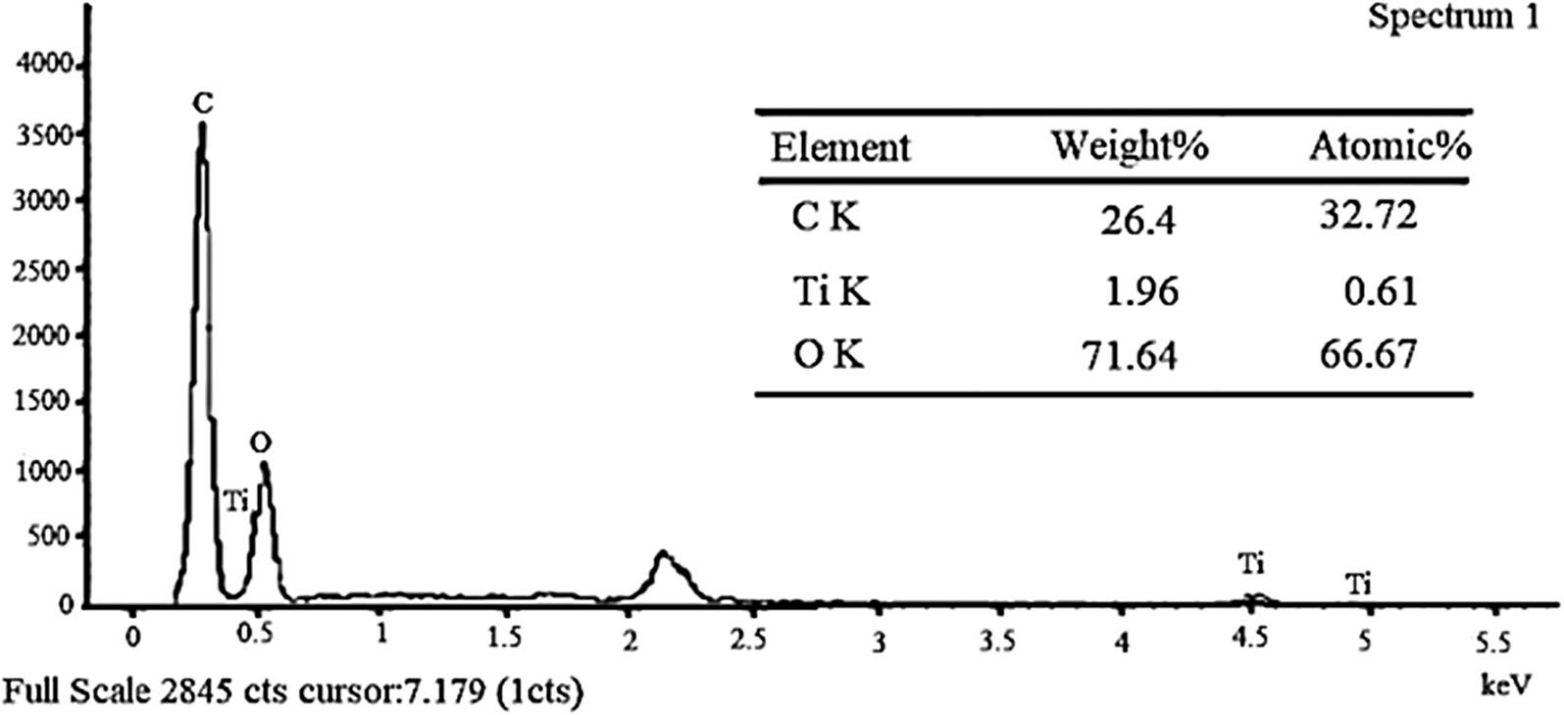
EDX analysis of SL/TiO_2_-1.0 composite

The element composition and distribution in the composites were investigated by EDX analysis. The EDX spectrum for KL/TiO_2_-1.0 and SL/TiO_2_-1.0 are shown in Figs. 13 and 14 respectively. Both composites were composed of the expected elements such as carbon, oxygen, and titanium. The carbon and oxygen can be assigned to lignin while titanium and oxygen can be ascribed to TiO_2_. It further indicates that TiO_2_ has been incorporated into lignin. The presence of sulfur in KL/TiO_2_-1.0 is owning to the hydrosulfide anions which are derived from the kraft pulping process [10].

### Relative production of hydroxyl radicals

**Fig. 15:**
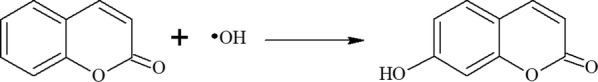
The photo-oxidation of coumarin to 7-hydroxycoumarin [24]

The relative production of hydroxyl radicals by kraft lignin, soda lignin, KL/TiO_2_-1.0, SL/TiO_2_ and anatase TiO_2_ were investigated through the photo-oxidation of coumarin to 7-hydroxycoumarin as a test reaction under the sunlight exposure. Coumarin reacts directly with hydroxyl radical to produce a fluorescing compound 7-hydroxycoumarin which can be detected by spectrofluorometer as depicted in Fig. 15. The highly fluorescent 7-hydroxycoumarin can be detected at 460 nm in the fluorescence spectrum [23].

### Comparison between lignin/TiO_2_ composite and pure anatase TiO_2_

**Fig. 16:**
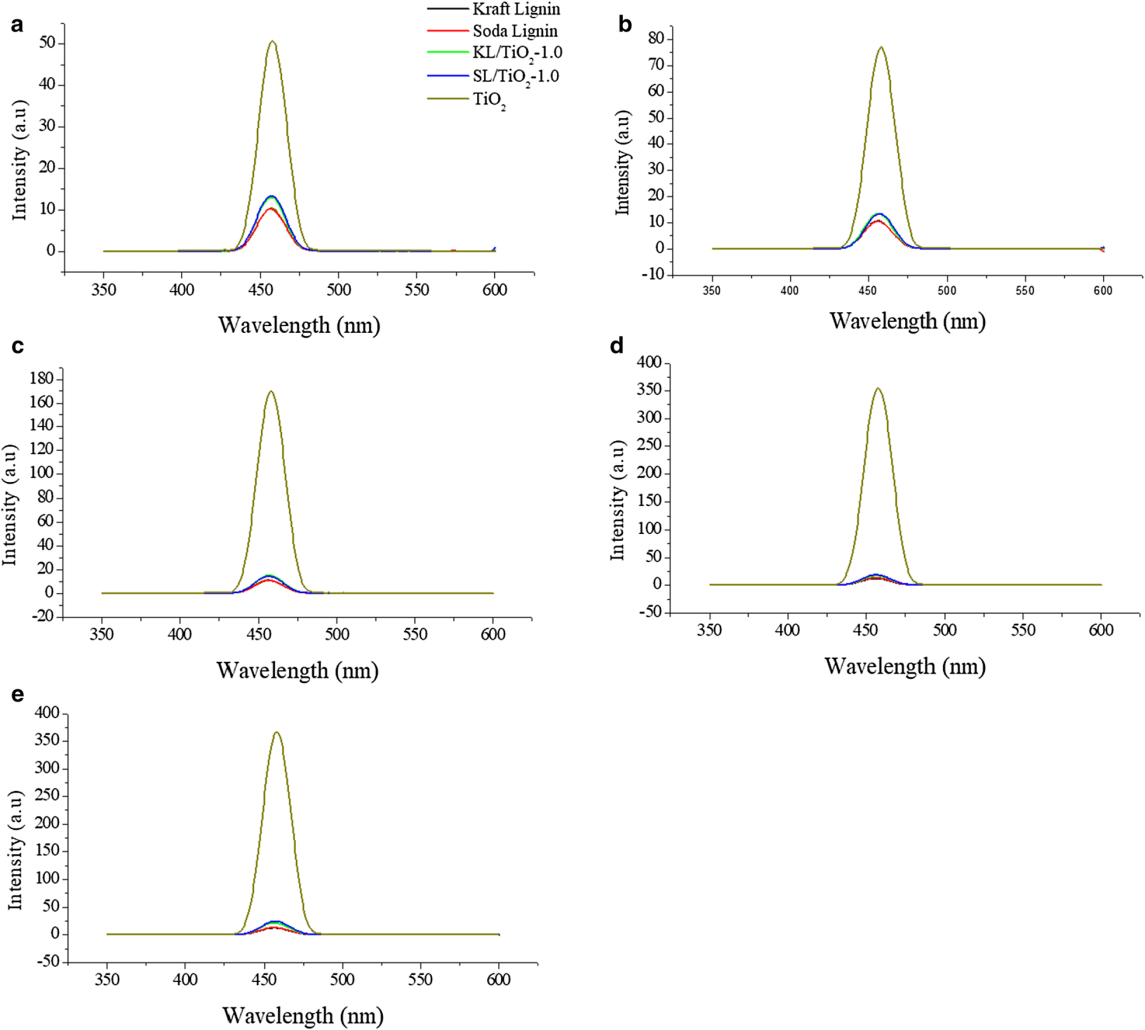
Fluorescence spectra of coumarin solution with kraft lignin, soda lignin, KL/TiO_2_-1.0, SL/TiO_2_-1.0 and TiO_2_ after being irradiated under sunlight for **a** 20 min, **b** 40 min, **c** 60 min, **d** 80 min, **e** 100 min

**Fig. 17:**
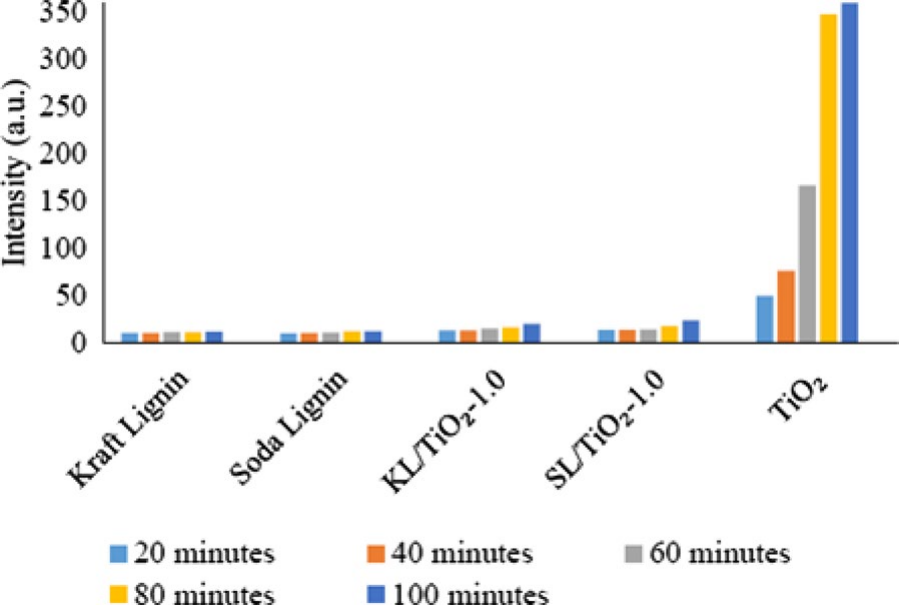
Fluorescence intensity of coumarin solution with kraft lignin, soda lignin, KL/TiO_2_-1.0, SL/TiO_2_-1.0 and TiO_2_ at 460 nm against sunlight illumination time

Fluorescence spectra of the coumarin solution irradiated under sunlight in the presence of kraft lignin, soda lignin, KL/TiO_2_-1.0, SL/TiO_2_-1.0, and anatase TiO_2_ are shown in Fig. 16. As shown in Figs. 16a–e and 17, coumarin solution in the presence of TiO_2_ exhibited the highest emission peak intensity throughout the experiment indicating the highest concentration of hydroxyl radicals being produced followed by SL/TiO_2_-1.0 and KL/TiO_2_-1.0. This can be attributed to the role of lignin as radical scavenger or antioxidant. Under the same sunlight irradiation, the peak intensity of coumarin solution in the presence of soda lignin and kraft lignin remained at the same level over irradiation time. This can be explained that both soda and kraft lignins possess no photocatalytic activity under sunlight irradiation. The result also indicated that the lignin does not completely quench the photocatalytic activity of TiO_2_ since hydroxyl radical generations still occur in the presence of both composites. The quenching process takes place when hydrogen atoms were abstracted from phenolic hydroxyl group by hydroxyl radical to form phenoxyl radical as proposed by Barclay et al. [24]. The presence of phenolic hydroxyl group in soda and kraft lignins has been proved by the UV and ^13^C NMR analyses. Moreover, according to the FTIR and ^13^C NMR spectra, both OPEFB lignins contained two main phenylpropanoid units which were guaiacyl (G) and syringyl (S). The methoxyl groups at the ortho position in G and S units aid in stabilizing phenoxyl radicals by resonance and hindering them from propagation. Besides, the interaction between the hydroxyl groups of phenolic compounds and the π-electrons of the benzene ring allows the phenolic compounds to produce radicals that can be stabilized by extended delocalization. Thus, the phenoxyl radical produced has much greater chemical stability than the initial radical.

### Comparison between types of lignin

**Fig. 18:**
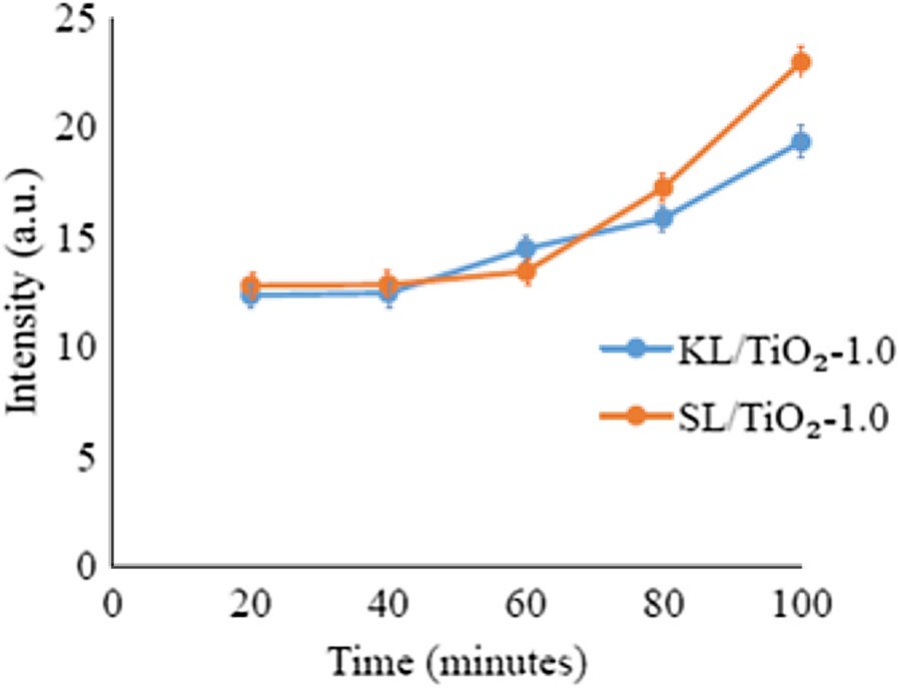
Fluorescence intensity of coumarin solution with KL/TiO_2_-1.0 and SL/TiO_2_-1.0 at 460 nm against sunlight illumination time

Figure 18 shows the fluorescence intensity of coumarin solution with KL/TiO_2_-1.0 and SL/TiO_2_-1.0 at 460 nm. The fluorescence intensity of both coumarin solutions increased with sunlight irradiation time. However, the fluorescence intensity of the solution with SL/TiO_2_-1.0 was higher than that of. KL/TiO_2_-1.0. The result indicates that kraft lignin has better hydroxyl radical quenching performance than soda lignin. This is highly possible due to higher phenolic hydroxyl group content of kraft lignin as mentioned in the UV and ^13^C NMR analysis. The higher phenolic hydroxyl content is caused by the severe process of kraft pulping which lead to extensive depolymerization of lignin such as cleavage of alkyl-aryl ether linkages. The hydroxide and hydrosulfide anions reacted with lignin causing the polymer to break down into smaller molecular weights fragments during the kraft pulping process and eventually the formation of new free phenolic hydroxyl group [10]. Hence, the low molecular weight fraction of kraft lignin possessed more non-etherified phenolic hydroxyl group than the high molecular weight fraction of soda lignin. It was suggested that the kraft lignin with higher content of non-etherified phenolic hydroxyl groups allows more hydroxyl radicals to be neutralized by the hydrogen atom abstraction from phenolic hydroxyl groups.

### Comparison between amounts of lignin

Since kraft lignin showed better performance in quenching hydroxyl radicals than soda lignin, it was used to prepare three other composites with different amount of lignin.

**Fig. 19:**
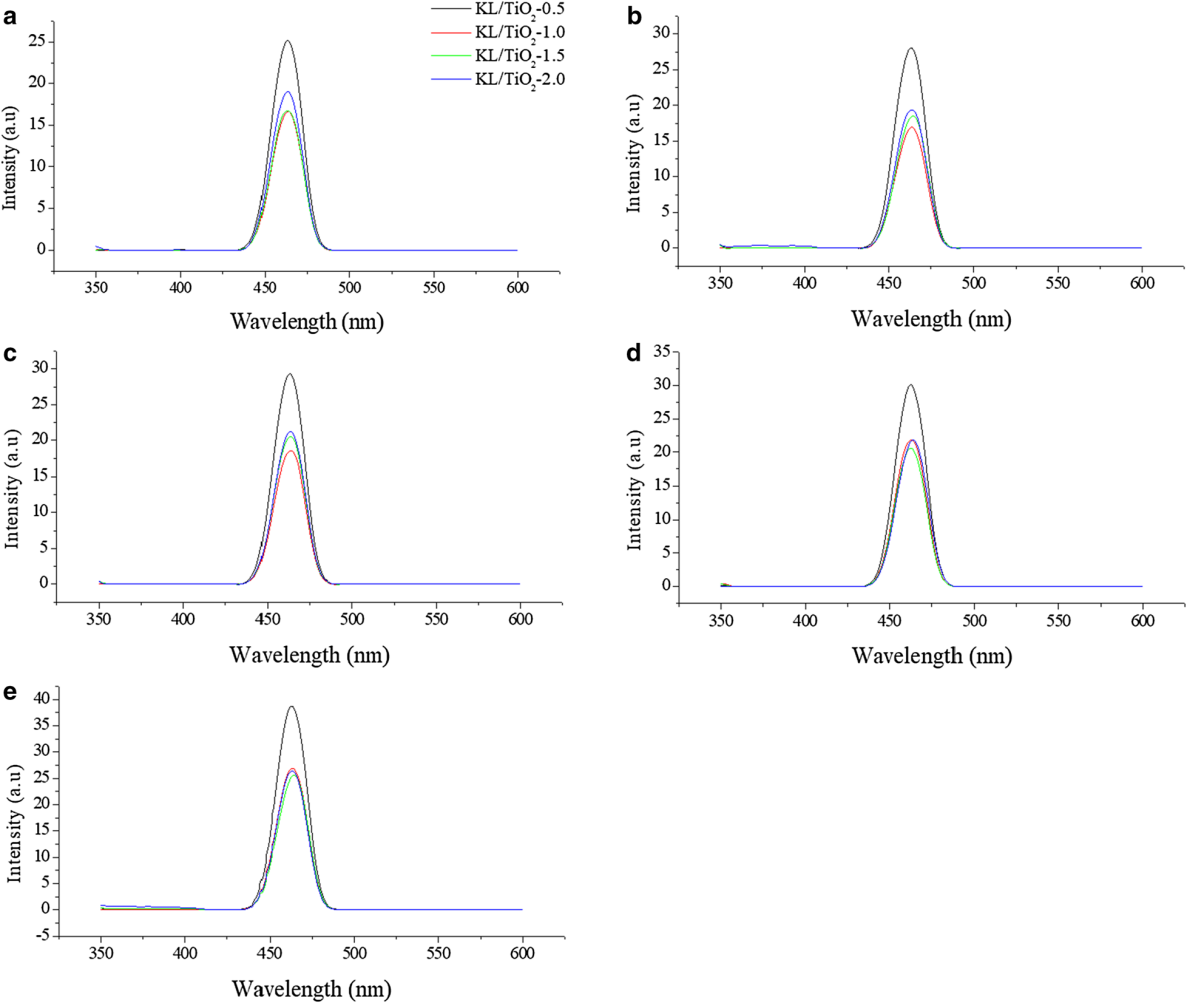
Fluorescence spectra of coumarin solution with KL/TiO_2_-0.5, KL/TiO_2_-1.0, KL/TiO_2_-1.5 and KL/TiO_2_-2.0 after being irradiated under sunlight for **a** 20 min, **b** 40 min, **c** 60 min, **d** 80 min, **e** 100 min

**Fig. 20:**
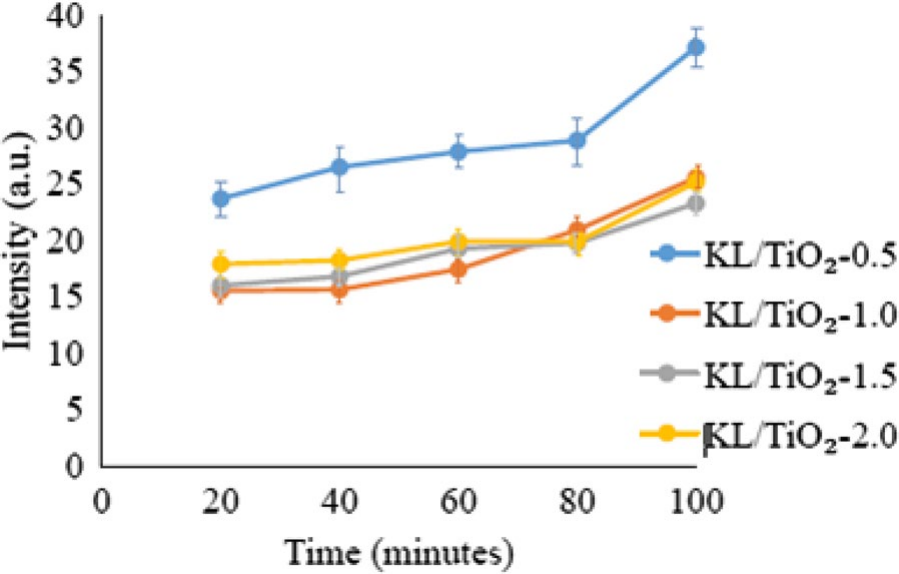
Fluorescence intensity of coumarin solution in the presence of KL/TiO_2_-0.5, KL/TiO_2_-1.0, KL/TiO_2_-1.5 and KL/TiO_2_-2.0 at 460 nm against sunlight illumination time

Figures 19a–e and 20 show that fluorescence intensity of coumarin solution in the presence of KL/TiO_2_-0.5 was the highest throughout the experiment compared to the solutions with other three composites. This may be due to the low amount of lignin used. The amount of lignin is not sufficient to form a neat coating layer around TiO_2_ particles and quench the hydroxyl radicals generated. According to Fig. 20, there was not much difference in the fluorescence intensities of the coumarin solutions with KL/TiO_2_-1.0, KL/TiO_2_-1.5 and KL/TiO_2_-2.0 under the same sunlight illumination. It was suggested that 1.0 of lignin is enough to cover TiO_2_ particles and quench hydroxyl radicals before they diffuse away from TiO_2_ which may cause damages to biomolecules or other sunscreen ingredients. Besides, the excess amount of lignin will also make the color of composite becomes darker which is unfavorable for the appearance of sunscreens. Although the fluorescent intensities of all the three coumarin solution increased slightly over the sunlight irradiation time, it does not mean that the lignin lost the capability to capture the free radicals. It could be attributed to the adsorption of coumarin molecules on vacant sites available on the surface of lignin. This will increase the competition between coumarin molecules and lignin in reacting with hydroxyl radicals. Thus, more coumarin adsorbed on the lignin surface over illumination time and produced more 7-hydroxycoumarin which lead to increasing of fluorescence intensity.

## Conclusion

In this study, lignin/TiO_2_ composites were successfully synthesized by using kraft lignin and soda lignin. This was confirmed by the results of FTIR, XRD, SEM and EDX analysis. The kraft lignin/TiO_2_ composite exhibited lowest fluorescence intensity compared to pure anatase TiO_2_ and soda lignin/TiO_2_ composite which indirectly corresponds to the lowest hydroxyl radical production. This is mainly due to the higher phenolic hydroxyl content of kraft lignin which has been proven by UV and ^13^C NMR analysis. The higher phenolic hydroxyl content provides more hydrogen atoms to quench the activity of the hydroxyl radicals. The excess amount of lignin did not improve the radical scavenging activity but gave a darker color of the composite which is unfavorable for sunscreens and cosmetic products.

## Abbreviations

TiO2: titanium II dioxide
OPEFB: oil palm empty fruit bunch
KL: kraft lignin
SL: soda lignin
